# Single Nucleotide Polymorphism rs1801516 in Ataxia Telangiectasia-Mutated Gene Predicts Late Fibrosis in Cancer Patients After Radiotherapy: A PRISMA-Compliant Systematic Review and Meta-Analysis

**DOI:** 10.1097/MD.0000000000003267

**Published:** 2016-04-08

**Authors:** Yuyu Zhang, Ziling Liu, Mengmeng Wang, Huimin Tian, Keju Su, Jiuwei Cui, Lihua Dong, Fujun Han

**Affiliations:** From the Department of Radiation Oncology (YZ, LD) and Cancer Center (ZL, MW, HT, KS, JC, FH), The First Hospital of Jilin University, Changchun, China.

## Abstract

Studies on associations between ataxia telangiectasia-mutated (ATM) polymorphisms and late radiotherapy-induced adverse events vary in clinical settings, and the results are inconsistent.

We conducted the first meta-analysis following Preferred Reporting Items for Systematic Reviews and Meta-Analyses (PRISMA) guidelines to investigate the role of the ATM polymorphism rs1801516 in the development of radiotherapy-induced late fibrosis.

We searched PubMed, Embase, Web of Science, and Chinese National Knowledge Infrastructure databases to identify studies that investigated the effect of the ATM polymorphism rs1801516 on radiotherapy-induced late fibrosis before September 8, 2015. Summary odds ratios (ORs) and the corresponding 95% confidence intervals (CIs) were used to assess the association between late fibrosis and the rs1801516 polymorphism. Subgroup analyses were conducted to evaluate the influence of clinical features on the genetic association. Tests of interaction were used to compare differences in the effect estimates between subgroups.

The overall meta-analysis of 2000 patients from 9 studies showed that the minor allele of the rs1801516 polymorphism was associated with a significantly increased risk of developing late fibrosis (OR = 1.78, 95% CI: 1.07, 2.94), with high between-study heterogeneity (I^2^ = 66.6%, *P* = 0.002). In subgroup analyses, we identified that the incidence of late fibrosis was a major source of heterogeneity across studies. The OR for patients with a high incidence of late fibrosis was 3.19 (95% CI: 1.86, 5.47), in contrast to 1.09 (95% CI: 1.01, 1.17) for those with a low incidence. There was a significant difference in the effect estimates between the 2 subgroups (ratio of OR = 2.94, 95% CI 1.70, 5.08, *P* = 0.031).

This meta-analysis supported previously reported effect of the ATM polymorphism rs1801516 on radiotherapy-induced late fibrosis. This finding encouraged further researches to identify more genetic polymorphisms that were predictive for radiotherapy-induced adverse events. In addition, we showed that the inconsistency of the associations seen in these studies might be related to variations in the incidence of late fibrosis in the patients. This suggested that future studies should consider the incidence of radiotherapy-induced adverse events when investigating radiosensitivity signature genes.

## INTRODUCTION

Radiotherapy-induced late adverse events cause a substantial decrease in quality of life, and are a major limiting factor in radiotherapy regimens. For patients treated with radiotherapy, the incidence of late adverse events increases as the radiation dose increases and as the follow-up time increases.^1,2^ There is a substantial interindividual variation in the extent of late adverse events even for patients who received similar or identical treatment protocols.^3^ A hypothesis thus arises that most of the individual differences are an inherited trait dependent on genetic background such as single nucleotide polymorphisms (SNPs).^4^ The discovery and application of biomarkers that incorporate with traditional dosimetry and clinical determinants can largely help to tailor radiotherapy to maximize efficacy and minimize adverse events.

A substantial amount of work has been performed over the past decade in an effort to identify SNPs that are associated with the development of normal tissue injuries after radiotherapy.^4^ The first gene that has received significant attention is the ataxia telangiectasia-mutated (ATM) gene.^5^ The product encoded by the ATM gene functions primarily as a protein kinase involved in cellular stress responses, cell cycle checkpoint control, and DNA repair.^6^ The ATM protein plays a central role in mediating the cellular response to radiation induced DNA damage such as double-stranded breaks.^7^ Patients with the disease of ataxia telangiectasia suffer from severe and devastating responses to ionizing radiotherapy.^5^

Our previous study indicated that the SNP most frequently studied in the ATM gene was rs1801516,^8^ also known as G5557A. rs1801516, a substitution of asparagine for aspartic acid at amino acid position 1853, is located in ATM exon 39.^5^ The functional impact of this polymorphism is unclear.^5^ A recent meta-analysis^9^ showed no significant association between the rs1801516 polymorphism and radiotherapy-induced adverse events in general. However, the studied adverse events included a variety of different clinical endpoints, involving a variety of different pathological mechanisms. In addition, this meta-analysis showed high unexplained between-study heterogeneity. Therefore, we had a concern about the rationale for combining all clinical endpoints. Another concern for this meta-analysis was an incomplete coverage of studies, including some large studies with appropriate statistical power.^10,11^ It is thus unclear whether there is a role of the rs1801516 polymorphism in the development of radiotherapy-induced adverse events. We have found that late fibrosis was the most frequently studied late adverse events (data unpublished). To address this issue, we conducted a meta-analysis of the rs1801516 polymorphism with a single clinical endpoint—late subcutaneous fibrosis.

## MATERIALS AND METHODS

This meta-analysis was conducted according to the Preferred Reporting Items for Systematic Reviews and Meta-Analyses (PRISMA) guidelines.^12^

### Ethics

Ethical approval was not necessary because this study was a systematic review and meta-analysis.

### Selection Criteria

**FIGURE 1:**
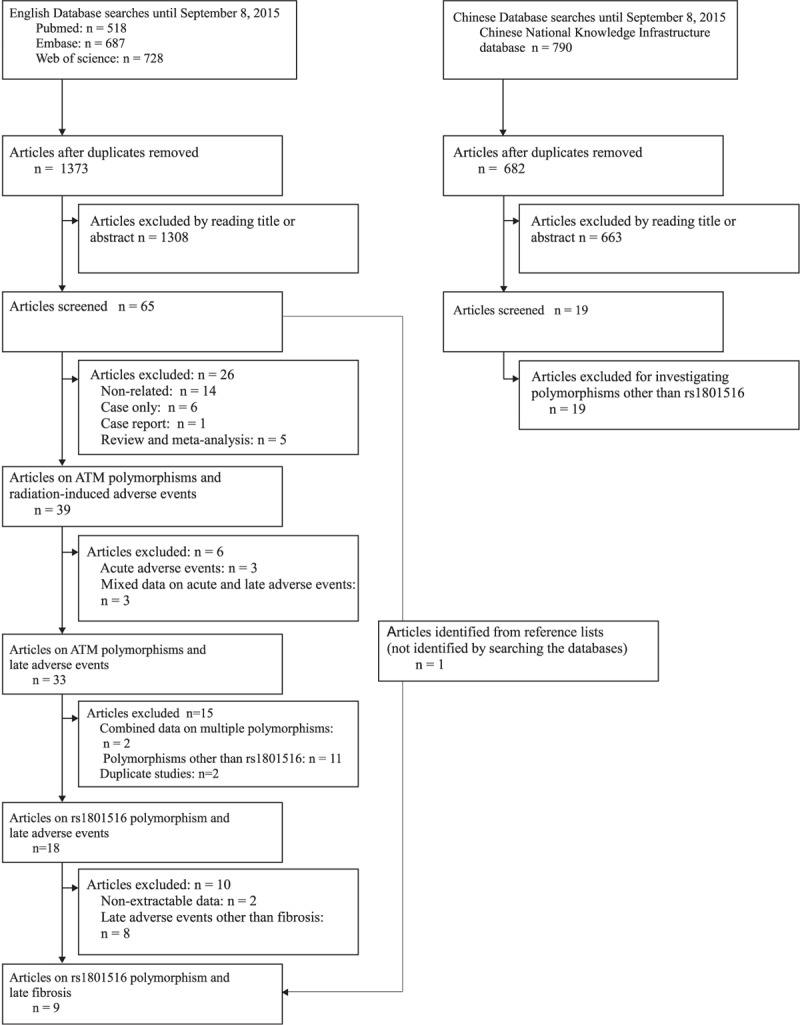
Flow chart for the process of selecting eligible studies.

Radiotherapy-induced late adverse events included a variety of different clinical endpoints, leading to potential heterogeneity in the analysis of the association between genetic polymorphisms and radiotherapy-induced late adverse events. We had found that late fibrosis was the most frequently studied late adverse events (data unpublished and Figure 1). Therefore, we focused on a single clinical endpoint—late fibrosis, to minimize the influence of potential confounding variables in this meta-analysis.

Studies that investigated the association between the ATM polymorphism rs1801516 and late fibrosis in patients of all cancer sites were included. No language restrictions were applied. If different studies reported on the same sample, only the most complete information was included. Case reports, editorials, meta-analyses, and review articles were excluded. Studies that reported combined data for acute and late radiotherapy-induced adverse events were excluded.

### Literature Search

A systematic literature search before September 8, 2015 was conducted in Electronic databases (PubMed, Web of Science, Embase, and Chinese National Knowledge Infrastructure [including China Doctoral/Master Dissertation Full-Text Database, China Academic Journals Full-Text Database, Century Journals Project, China Proceedings of Conference Full-Text Database]). The following search terms were used: (radiation OR radiotherapy) AND (ATM OR ataxia-telangiectasia mutated) AND (polymorphism^∗^ OR variant^∗^ OR mutant^∗^ OR genotype^∗^). References from the relevant articles or reviews were also searched for additional studies. In addition, we searched the Internet (www.google.com) for unpublished data. To avoid the use of different names for the same polymorphism, we included all surrogates of the rs1801516 polymorphism in this meta-analysis, including rs17503060, rs52821794, rs60879649 (http://www.ncbi.nlm.nih.gov/snp/), and rs4988023.^10^

### Data Collection

Two authors independently extracted the following data: first author, year of publication, period of radiotherapy, country of origin, minor allele frequency in patients, Hardy–Weinberg equilibrium (HWE) in patients, sample size, incidence of late fibrosis, cancer site, length of follow-up, study type (cohort and case–control), number of genotyped cases and controls, and results on the association between the rs1801516 polymorphism and late fibrosis. Data on treatment- and injury-related factors were also collected from each study. Treatment-related factors included surgery and radiotherapy (e.g., total dose, dose per fraction). Injury-related factors included clinical endpoint and diagnosis criterion. Study authors were contacted when there was insufficient information to determine the relationship between the rs1801516 polymorphism and late fibrosis. Disagreement was resolved by discussion between authors.

### Procedure

Study quality was assessed on the basis of HWE in patients (yes/no), and sample size (large/small), adhering to the established criteria.^13^ It should be noted that deviation from HWE in patients might not point to a sampling bias or mistyping of genotypes, but could be an evidence of association between the genotype and the disease.^14^ Two authors independently evaluated the quality of each study, with discrepancies resolved during a consensus meeting. Because a summary quality score can lead to bias in the results of a meta-analysis,^15–17^ it was not used to weigh the contribution of each study to the meta-analysis. Instead, the study quality was used as a stratification factor in the subgroup analysis to evaluate its influence on the effect size.^17,18^

A single primary meta-analysis was performed on all datasets. Subgroup meta-analyses were conducted based on prespecified clinical features, including sample size, ethnicity, HWE in patients, cancer site, incidence of late fibrosis, and period of follow-up. We aimed at determining whether the result of the primary meta-analysis was stable or dependent on the clinical features of the included studies. Sensitivity analysis was conducted by excluding 1 study at a time and analyzing the remaining ones to explore whether the result was influenced by a particular study.

### Statistical Analysis

We performed the appropriate goodness-of-fit χ^2^ test to assess deviation from HWE. Because homozygotes and heterozygotes of the rs1801516 polymorphism were grouped together (dominant model) in most included studies, we conducted meta-analyses only under the dominant model. Odds ratios (ORs) and the 95% confidence intervals (CIs) were used to assess the strength of the association between the rs1801516 polymorphism and late fibrosis. The statistical significance of the ORs was evaluated by using the Z test. Between-study heterogeneity was evaluated by using the Cochrane Q test and the I^2^ statistic. The random-effects model (DerSimonian and Laird method)^19^ was used to calculate ORs when the *P* value of the Cochrane Q test was <0.10 or the I^2^ value was >50%^20,21^; otherwise, we used the fixed-effects model. We used the test of interaction proposed by Altman et al^22^ to compare differences in effect estimates between subgroups. The above statistical analyses were performed by using Stata, version 12, software (StataCorp LP, College Station, TX) with 2-sided *P* values. Statistical significance was defined as a *P* value of <0.05.

## RESULTS

The initial search for 3 English databases (PubMed, Embase, and Web of Science) yielded 1373 studies dated until September 8, 2015. Of these, 1308 studies were irrelevant by title or abstract reading. After full text reading, 39 studies were found to investigate the association between ATM polymorphisms and radiation-induced adverse events. We then excluded studies that reported acute adverse events (n = 3), mixed data on acute and late adverse events (n = 3), combined data on multiple polymorphisms (n = 2), polymorphisms other than rs1801516 (n = 11), and duplicate samples (n = 2). 18 studies were identified to investigate the rs1801516 polymorphism and the risk of developing late adverse events. After further exclusion of studies with nonextractable data (n = 2) and studies on late adverse events other than fibrosis (n = 8), 8 published studies^5,10,11,23–27^ were identified to meet the inclusion criteria. In addition, 1 study^28^ was identified from the references of a review article.^29^ Our search on Chinese National Knowledge Infrastructure database identified no study that met the inclusion criteria (possibly due to a low minor allele frequency of <0.05 in Asian).^30,31^ No unpublished data were identified using Internet search (www.google.com). As a result, a total of 9 studies were eligible for this meta-analysis (Figure 1). The study characteristics were presented in Table 1.

**TABLE 1:**
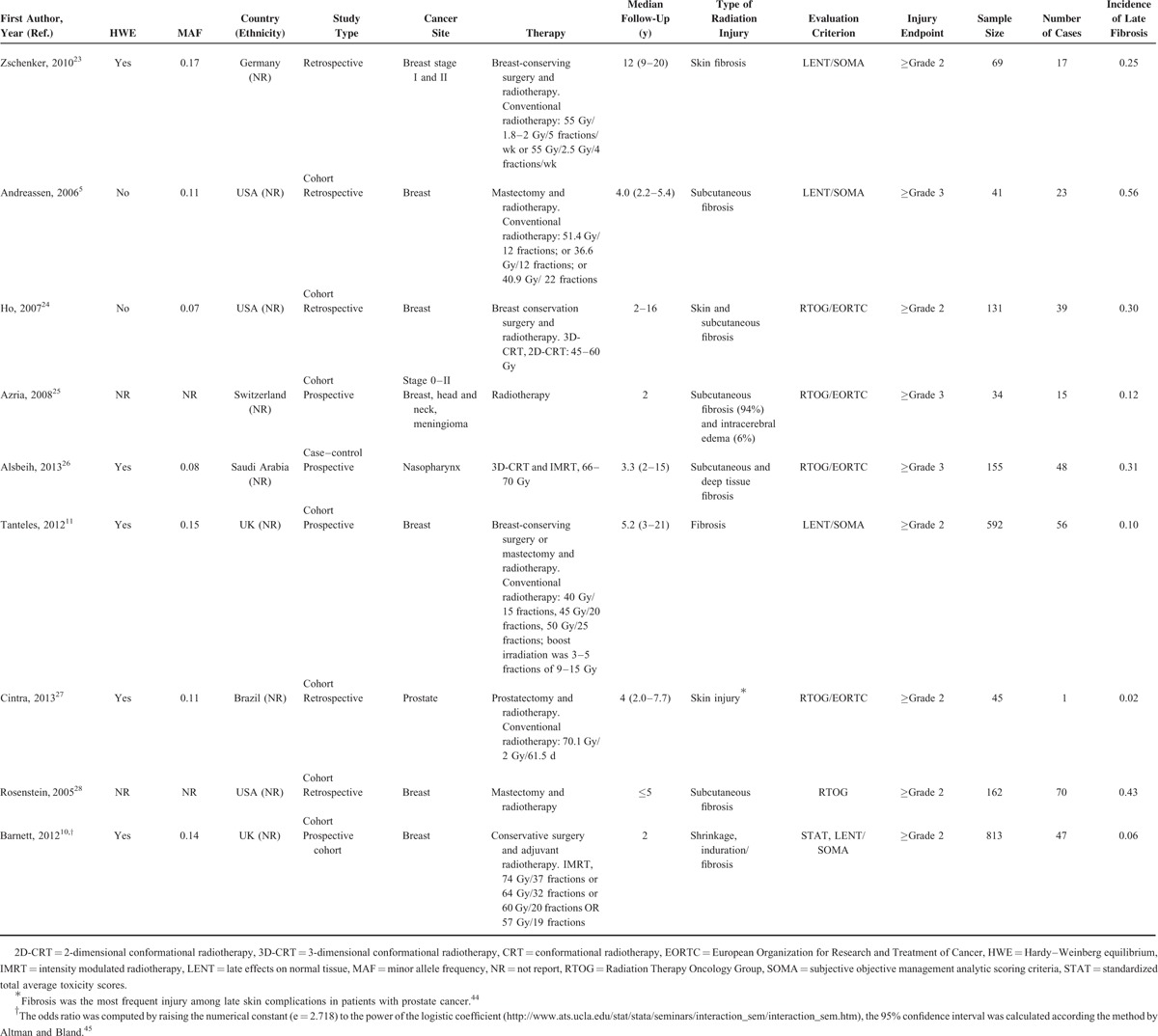
Description of Studies Included in the Meta-Analysis of Ataxia Telangiectasia-Mutated rs1801516 Polymorphism and the Risk of Late Fibrosis

These studies were published between 2005 and 2013. 3 out of 9 studies were conducted in the United States,^5,24,28^ 2 in United Kingdom,^10,11^ 1 in Switzerland,^25^ 1 in Saudi Arabia,^26^ 1 in Brazil,^27^ and 1 in Germany.^23^ None of these studies stated the ethnicity of the participants (therefore, an assessment of the effects of population stratification was not conducted in the subsequent analyses). For each study, the endpoint was selected which was judged to reflect late fibrosis most closely. The cut-off to differentiate cases from controls was grade 2 in 5 studies,^10,23,24,26,27^ and grade 3 in 2 studies.^5,25^ The number of patients within each grade was given in 2 studies.^11,28^ Therefore, a grade 2 cut-off was set for differentiating cases from controls in this meta-analysis.

**FIGURE 2:**
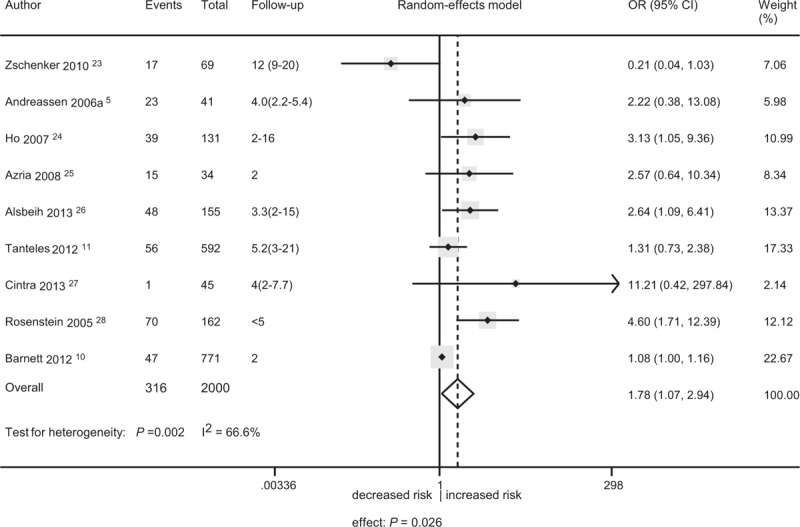
Forest plot for the association between the ataxia telangiectasia-mutated polymorphism rs1801516 and the risk of late fibrosis. CI = confidence interval, OR = odds ratio.

Overall, 9 studies with 2000 patients was included in the primary meta-analysis. There was a significant association between the rs1801516 polymorphism and late fibrosis (OR = 1.78, 95% CI: 1.07, 2.94; *P* = 0.026), with high between-study heterogeneity (I^2^ = 66.6%; *P* = 0.002) (Figure 2). The leave-one-out sensitivity analysis showed that no single study dramatically influenced the result of this meta-analysis, indicating that the result was reliable. The study by Zschenker et al^23^ showed that the rs1801516 minor allele carriers had a decreased risk of developing late fibrosis. In contrast, the other studies consistently showed an increased risk (significant in 2 studies^26,28^ and nonsignificant in 6 studies)^5,10,11,24,25,27^ for the minor allele carriers. Notably, the study by Zschenker et al^23^ had the longest median follow-up of 12 years compared with a median follow-up of no more than 5.2 years in the other studies.^5,10,11,24–28^ The subgroup of long-term follow-up (n = 69), including only the study by Zschenker et al,^23^ had an OR of 0.21 (95% CI: 0.04, 1.03). The genetic effect for studies with short-term follow-up (n = 1931) was significant (OR = 2.04, 95% CI: 1.24, 3.36; *P* = 0.005; I^2^ = 64.5%, *P* = 0.006). The effect sizes were significantly different (ratio of odds ratio [ROR] = 0.10, 95% CI: 0.02, 0.56; *P* = 0.009). Due to the small sample size of the subgroup of long-term follow-up, the result of the interaction test should be considered as exploratory only.

Because the Zschenker et al study^23^ was different from the other studies in the follow-up period and the genetic effect on late fibrosis, it was considered as an outlier. Therefore, this study^23^ was excluded from the subsequent subgroup meta-analyses. Because sample size is a continuum, distinction between large and small is inevitably arbitrary. To minimize the effect of subjective interpretation of the data, the cut-off for differentiating a large from a small study was based on the median of the sample sizes across all included studies (n = 143). Similarly, for the incidence of late fibrosis, the cut-off for differentiating high from low incidence was also based on the median of the incidences across all included studies (21%). When subgroup analyses were conducted based on cancer site, sample size, definition of cases, and HWE in participants, high between-study heterogeneity was observed (Table 2). This suggested that the above-mentioned covariates did not contribute to the heterogeneity. We identified the source of heterogeneity when the studies were divided based on the incidence of late fibrosis: the participants with a high incidence had larger genetic effect (OR = 3.19, 95% CI: 1.86, 5.47, *P* < 0.001; I^2^ = 0.0%; *P* = 0.836) compared versus the participants with a low incidence (OR = 1.09, 95% CI: 1.01, 1.17, *P* = 0.026; I^2^ = 21.9%; *P* = 0.279). The effect sizes were significantly different (ROR = 2.94, 95% CI: 1.70, 5.08; *P* = 0.031).

**TABLE 2:**
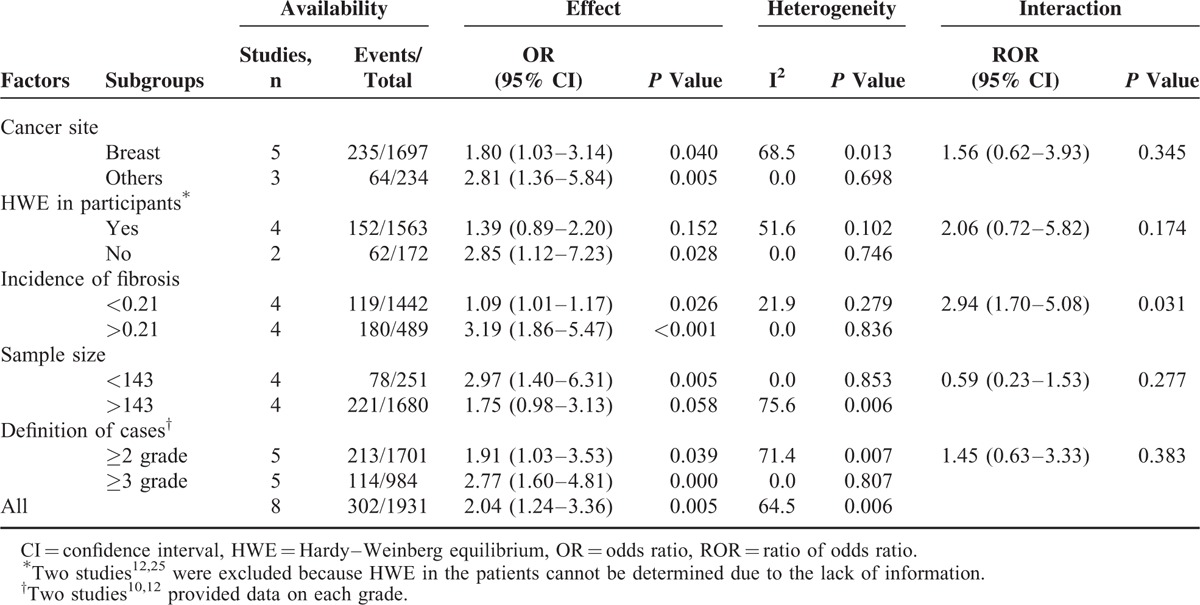
Subgroup Analyses for the Genetic Effect of the Ataxia Telangiectasia-Mutated Polymorphism rs1801516 on the Risk of Late Fibrosis

## DISCUSSION

Our meta-analysis of 2000 patients from 9 studies provided evidence of an association between the ATM polymorphism rs1801516 and the risk of developing late fibrosis in cancer patients after radiotherapy. The inconsistent association between late fibrosis and the rs1801516 polymorphism observed in some studies was probably attributable to variations in the follow-up period and the incidence of late fibrosis in the patients.

Our meta-analysis suggested that the length of follow-up might be a factor influencing the observed genetic effect of the rs1801516 polymorphism on late fibrosis in the patients. Late normal tissue injuries typically manifest years after radiotherapy, and this latency–response relationship may be fairly steep in clinical range, which means a small difference in latency can produce divergent outcomes.^2,32^ In addition, there is a possible existence of gene–survival interactions, which in turn may influence the genetic association. Therefore, for a reliable assessment, patient-to-patient variability due to genetic factors in late tissue injuries has to be evaluated in a small time span.^2,32^ In this meta-analysis, the included studies differed significantly in the length of follow-up, which was highly likely to generate heterogeneities. Indeed, the rs1801516 polymorphism was shown to be associated with an increased risk of late fibrosis in the subgroup of short-term follow-up, but with a trend in the opposite direction in the subgroup of long-term follow-up. The result of interaction tests supported a genuine difference in the effect sizes between the 2 groups. However, due to the relatively small number of studies included in the subgroup of long-term follow-up, and this finding should be treated with caution.

An increased risk (significant or nonsignificant) of late fibrosis was consistently shown for the minor allele carriers of the rs1801516 genotype across all studies with short-term follow-up (Figure 2). However, the included studies displayed a marked clinical variability in terms of cancer site, treatment strategy, co-morbidity, and definition of cases. The variability was equivalent to adding “noise” to the analyses, leading to any true association becoming less significant.^33^ Despite the variability, the association between the rs1801516 polymorphism and an increased risk (significant or nonsignificant) of late fibrosis was also consistently seen across different patient subgroups (Table 2). The extensive consistency provided optimal evidence of the credibility of an association.^34^ Furthermore, the credibility was strengthened by the clinical variability. Therefore, our meta-analysis gave strong evidence of an association between the rs1801516 polymorphism and an increased risk of late fibrosis.

The genetic effect of the rs1801516 polymorphism on the development of late fibrosis was modified by the incidence of late fibrosis in the patients. This result was in agreement with a recent meta-analysis on the association between the rs1801516 polymorphism and acute radiotherapy-induced adverse events.^8^ Late injuries show a dose–effect relationship, which may be steep in clinical range. As a result, a small dose difference can cause a substantial interpatient variability in late injuries.^1,29^ It was therefore crucial to determine whether radiation dose was a source of heterogeneity across these studies. However, the radiation dose received by a normal tissue is not straightforward. For a meta-analysis, obtaining complete dose information in individual patients is generally not possible. Because there is a strong correlation between radiation dose and the incidence of late injuries,^35,36^ the incidence of late injuries can be used to estimate the radiation dose received by the normal tissue in the patients. Previous studies also demonstrated that an ideal population to identify genetic factors affecting radiosensitivity was patients with a high incidence of radiation injuries.^5,37^ Consistently, the results from both our subgroup analyses and interaction tests showed that the incidence of late fibrosis was the major source of heterogeneity across these studies.

There were a number of possible weaknesses in this meta-analysis. The sample size was small for some subgroup analyses (e.g., subgroup of nonbreast cancers), and the interpretation of these results should be taken carefully. This meta-analysis was based on summary data and not on individual patient data (IPD). An IPD-based meta-analysis is able to give an effect estimate that is adjusted for covariates.^38^ However, meta-anlayses based on summary data are often consistent with those based on IPD,^39^ and should not be viewed as “inferior.”^40^ Clearly, further analyses using IPD should be conducted to assess main genetic effects as well as interactions between covariates and genetic effects. The publication bias was not tested by the funnel plot due to heterogeneity observed in this meta-analysis, for tests to assess publication bias is unreliable when there is heterogeneity in the meta-analysis.^41^ However, small studies did not show significantly larger effects than large studies. In addition, according to the Venice criteria that had been developed to assess cumulative evidence of genetic associations, a small OR (OR < 1.15) might be vulnerable to biases (selective reporting biases, population stratification, and genotyping errors).^34,42,43^ Our primary meta-analysis showed an OR of 1.78, indicating that this genetic effect was not so vulnerable to biases. Except for the dominant model, other genetic models (i.e., recessive, additive, or homozygote) were not examined because of the limited information in the included studies. Therefore, the most appropriate genetic model for the genetic association could not be determined. None of the included studies reported subgroups based on tumor stage or tumor subtype, so sources of between-study heterogeneity were not able to be investigated by stratifying analyses accordingly. However, the results of our subgroup analyses showed that majority of the heterogeneity was explainable by the incidence of late fibrosis in the patients.

## CONCLUSION

After a decade of extensive research on this issue, this meta-analysis found strong evidence of an association between the ATM polymorphism rs1801516 and late fibrosis in patients after radiotherapy. This finding may be an important step toward personalized radiotherapy. Next, we will make an effort to obtain more genetic indicators, and our ultimate aim is to integrate genetic information to optimize radiotherapy. In addition, we found that the observed genetic effect of the rs1801516 polymorphism might be modified by the incidence of late fibrosis in patients. This suggested that future studies should consider incidence of radiotherapy-induced adverse events as a key factor when assessing radiosensitivity signature genes.
